# Attenuation, dispersion and nonlinearity effects in graphene-based waveguides

**DOI:** 10.3762/bjnano.6.125

**Published:** 2015-05-28

**Authors:** Almir Wirth Lima, João Cesar Moura Mota, Antonio Sergio Bezerra Sombra

**Affiliations:** 1 Laboratory of Telecommunications and Materials Science and Engineering, Fortaleza, Ceará, Brazil; 2 Department of Teleinformatics Engineering, DETI, Center of Technology, Federal University of Ceará, Fortaleza, Ceará, Brazil

**Keywords:** graphene, nanophotonics, waveguide

## Abstract

We simulated and analyzed in detail the behavior of ultrashort optical pulses, which are typically used in telecommunications, propagating through graphene-based nanoribbon waveguides. In this work, we showed the changes that occur in the Gaussian and hyperbolic secant input pulses due to the attenuation, high-order dispersive effects and nonlinear effects. We concluded that it is possible to control the shape of the output pulses with the value of the input signal power and the chemical potential of the graphene nanoribbon. We believe that the obtained results will be highly relevant since they can be applied to other nanophotonic devices, for example, filters, modulators, antennas, switches and other devices.

## Introduction

The production of smaller yet more simple and efficient electronic and photonic devices is the biggest concern of industry and commerce, given the increase in the user requirements for these devices. Meanwhile, the electrical and optical characteristics of graphene are attracting attention from researchers around the world. The plasmon technology on which this new material is based has enabled the development of nanoscale devices, which are also faster and more efficient than traditional devices found today. The nanoscale dimensions of graphene-based plasmonic devices allow for their integration into electronic and photonic integrated circuits.

Since there are many nanophotonic devices based on nanophotonics waveguides, this study was focused on the simulation and analysis of the attenuation, dispersion and nonlinear effects occurring in signals propagating through a graphene-based waveguide. We considered a graphene nanoribbon located between similar dielectric layers, as will be described further. Previous reports illustrated several details regarding these waveguides [1–7]; however, the attenuation, dispersion and nonlinear effects were not focused on in detail.

Given that graphene surface plasmon polaritons (GSPPs) are highly confined in graphene nanoribbons acting as waveguides, it is possible to integrate these waveguides into photonic integrated circuits (PICs). However, the nanoribbon width (*W*) strongly influences the mode behavior that propagates through these graphene nanoribbons. In this sense, the smaller the nanoribbon width, the lower the number of modes that are present in the waveguide. Previous studies showed that in a graphene nanoribbon of width <50 nm, there exists only a single mode (fundamental mode) [3]. However, due to finite-size effects, when *W* < 10 nm, the classical theory can no longer predict the behavior of GSPPs in a graphene nanoribbon [8].

Recent reports showed that it is possible to couple the radiation emitted by a transmitter (located above and to the side, but in the same plane of the graphene sheet) with the surface plasmons (SPs) present on a graphene sheet [9]. Other experiments showed the characteristics of GSPP guided modes, with a wavelength in air of 1.31 μm at a repetition rate of 2.5 Gbps, which propagated in a waveguide consisting of a graphene nanoribbon embedded in a dielectric medium. The dielectric layers above and below the graphene nanoribbon had the same refractive index [10].

The guided TM polarized GSPPs modes were excited through a polarization maintaining single mode fiber, by the use of the end-fire coupling method.

This paper is organized as follows: in the following section, we present the characteristics of waveguides composed of a single graphene nanoribbon. After the simulation and analysis is presented, the attenuation, dispersion and nonlinear effects occurring in a graphene-based waveguide are described in the Results and Discussion section. The last section summarizes the conclusions.

## Graphene-based waveguides features

The local conductivity of graphene, σ = σ_intra_ + σ_inter_, can be estimated by the Kubo formula, given by [11]:

[1]
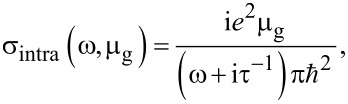


where σ_intra_ is the intraband conductivity (due to electron–photon scattering), μ_g_ is the chemical potential of graphene, *e* is the electron charge, ω is the angular frequency of the light in air and τ is the phenomenological scattering rate. Moreover, the interband conductivity (due to the electron transition) is given by [12]:

[2]
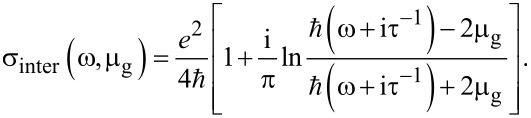


It is worth noting that Equation 1 and Equation 2 are valid for μ_g_ >> *k*_B_*T* and *T* = 300 K (room temperature), thus, *k*_B_*T* ≈ 26 meV.

The imaginary part of the conductivity determines the type of the polarized modes that can be supported in a graphene nanoribbon. For σ′′ > 0 (where σ′′ is the complex part of the conductivity of graphene), TM modes can be supported, while for σ′′ < 0, TE modes can be supported [13].

In the limit without collision between electrons (*T* = 0, *k*_B_*T*/μ_g_ = 0), the TM modes (s-polarization) and TE modes (p-polarization) are supported in graphene when 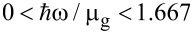 and 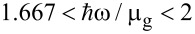, respectively. By numerical simulations it was proved that energy is absorbed or dissipated when 
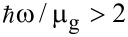
. However, if we consider a fixed graphene chemical potential, the temperature increase causes a finite damping, which is smaller for TM vs TE modes. This is because the real part of the conductivity undergoes more changes in the region where the TE modes are located [14].

Starting from Maxwell’s Equations, we arrive at the expression that relates the electric permittivity of a graphene nanoribbon to the angular frequency (ω), conductivity and graphene effective thickness (*t*) of the graphene nanoribbon, given as [15–16]:

[3]
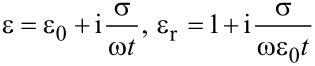


In a graphene nanoribbon embedded in a substrate with relative permittivity ε_r_, the TM modes are dominant. Considering the nonretarded regime (*q >>* ω/*c*, where *c* is the speed of light in air), the equation for the dispersion relation for graphene nanoribbons is given by [17]:

[4]
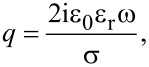


where ε_r_ is the relative permitivity in which the graphene nanoribbon is embedded.

The dispersion relation for graphene nanoribbons for TM modes propagating along a graphene/dielectric interface is also given by [18]:

[5]
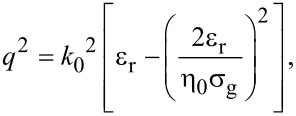


where η_0_ = 377 Ω is the air impedance and *k*_0_ = 2π/λ_0_. Similarly, the dispersion relation for the TE modes is given by:

[6]
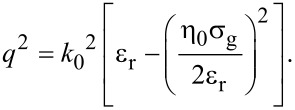


Since the wave vectors of the surface plasmons polaritons in a graphene/dielectric surface (GSPPs) have high values, we can conclude that these modes are very well confined in the graphene nanoribbon.

Previous studies have shown that GSPPs can only propagate in graphene nanoribbons when the plasmon energy corresponds to −iσ(ω)/ω*W*, where *W* is the width of the graphene nanoribbon [3].

As previously mentioned, in a graphene nanoribbon of width <50 nm, only a single mode (fundamental mode) exists [3]. On the other hand, due to the finite-size effects, when *W* < 10 nm, the classical theory can no longer determine the behavior of GSPPs in the graphene nanoribbon [8]. Hence, we primarily consider the regime 10 nm < *W* < 50 nm for our simulations.

**Figure 1:**
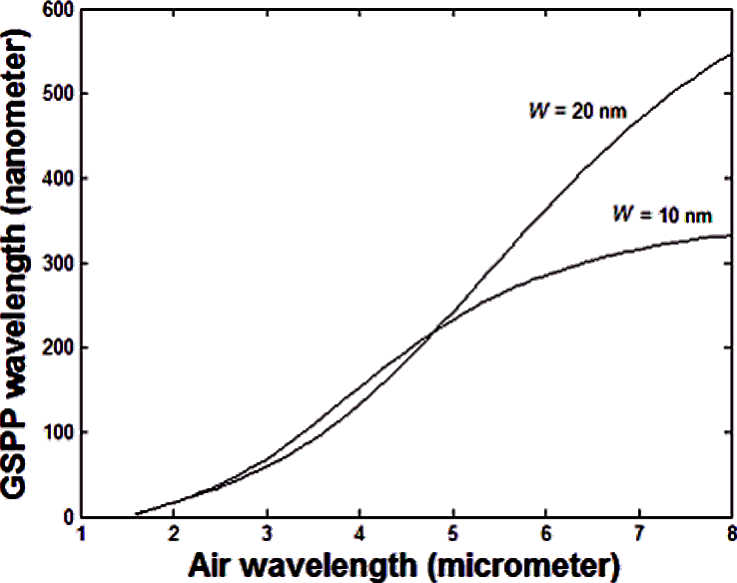
GSPP mode wavelengths as a function of the fundamental electromagnetic wavelength in the air for nanoribbon widths *W* = 10 nm and *W* = 20 nm; µ_g_ = 0.5 eV.

However, as we are interested in analyzing the behavior of nanophotonic waveguides with the smallest possible widths, we simulate these waveguides for widths *W* = 10 nm and *W =* 20 nm*.* Figure 1 shows the results for the wavelength of GSPPs modes as a function of the fundamental mode wavelength in air (before coupling to the SPs) for nanoribbon widths of *W =* 20 nm and *W =* 10 nm (μ_g_ = 0.5 eV).

From Figure 1 we can see that it is not possible to propagate GSPPs modes coupled to light with λ_0_ ≈< 1.55 μm in graphene nanoribbons with a width of 10 nm and 20 nm (µ_g_ = 0.5 eV). However, to work around this problem, we can increase the value of the graphene chemical potential. Note that the GSPP wavelengths for a graphene nanoribbon of width *W* = 20 nm are greater than the values for *W* = 10 nm, that is, the GSPPs modes in a narrower nanoribbons are more confined.

**Figure 2:**
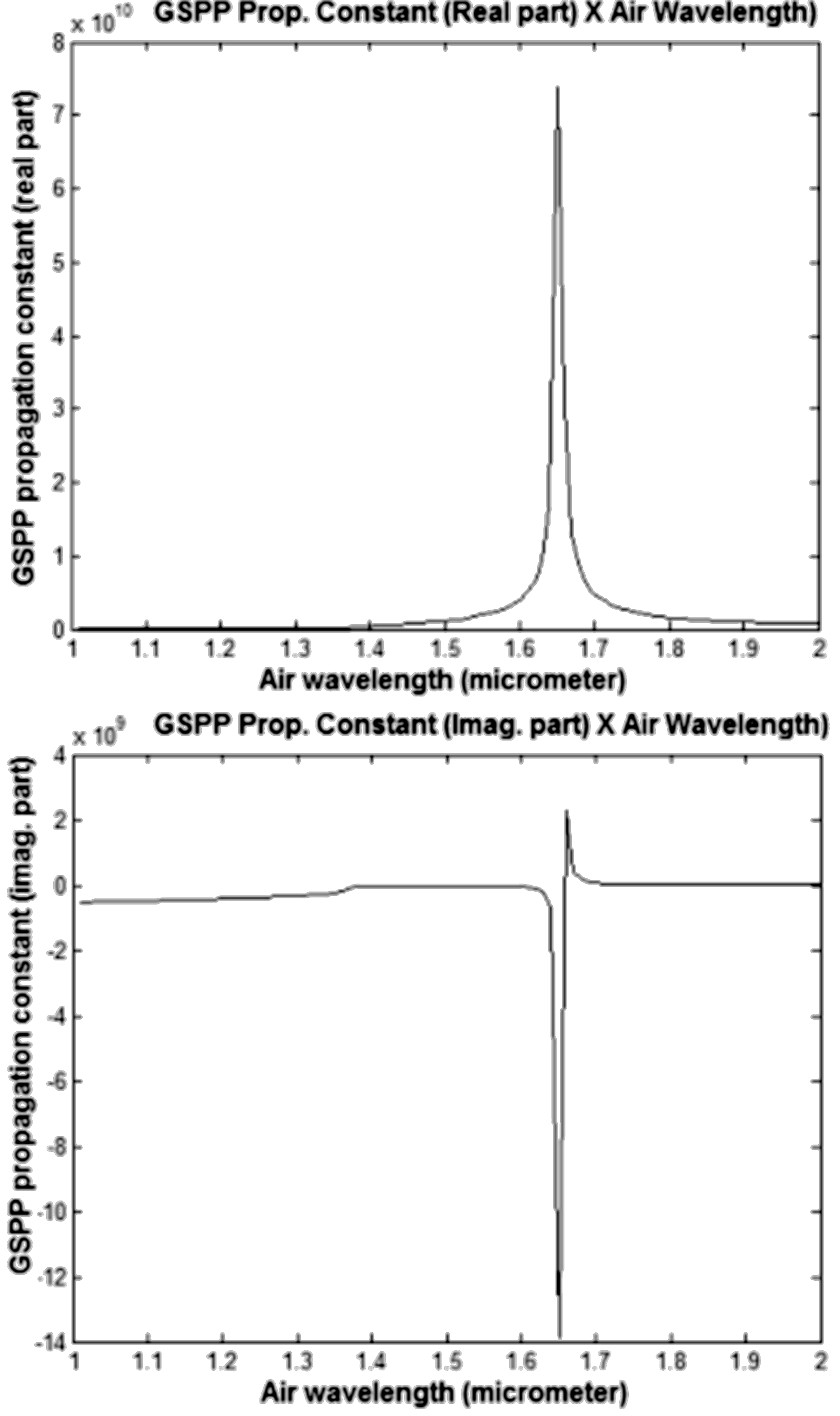
Wave vector (real and imaginary parts) for 1 μm < λ_0_ < 2 μm and μ_g_ = 0.45 eV.

To simplify our calculations, we considered graphene nanoribbons suspended in air. The real and imaginary parts of the GSPP wave vector as a function of the wavelength in air (before coupling to the SPs) for 1 μm < λ_0_ < 2 μm and μ_g_ = 0.45 eV are shown in Figure 2.

Taking into account that the propagation length of the GSPP mode (where the signal intensity drops to 1/*e* of its initial intensity) is given by *L*_p_ = Im(*q*)/2, where *q* is the wave vector of the GSPP TM modes, we can see from the lower part of Figure 2 that *L*_p_ remains constant for wavelengths up to 1.7 μm. Also note that in this wavelength range, the value of the ratio Re(*q*)/Im(*q*) = ωτ (where τ is the relaxation time) varies very little.

After the transfer of graphene onto a substrate, it should be free from wrinkles or distortions. However, the thermal SiO_2_ deposition process often results in high surface roughness, such that graphene on SiO_2_ shows no charge homogeneity along its surface [19].

Hexagonal boron nitride (h-BN, also known as white graphite) is a graphite isomorphic insulator in which the boron and nitrogen atoms occupy positions A and B in the Bernal structure. Therefore, the atomic structure of h-BN is similar (hexagonal) to the structure of graphene. It is worth noting that graphene on a layer of h-BN has a charge carrier mobility value and homogeneity of almost an order of magnitude better than on SiO_2_. Hence, graphene should be supported on a layer of h-BN, which in turn should be above (or below) the SiO_2_ substrate. Previous studies have defined the value of the mobility in graphene as μ_g_ = 60,000 cm^2^/Vs [20]. It is important to state that the dielectric properties of h-BN are similar to the dielectric properties of SiO_2_.

The surface roughness of the h-BN layer is much smaller than the surface roughness of SiO_2_, so that a graphene nanoribbon is better positioned on the surface of a h-BN layer. Methods for the deposition of a h-BN layer on SiO_2_/Si already exist, as detailed in [19].

**Figure 3:**
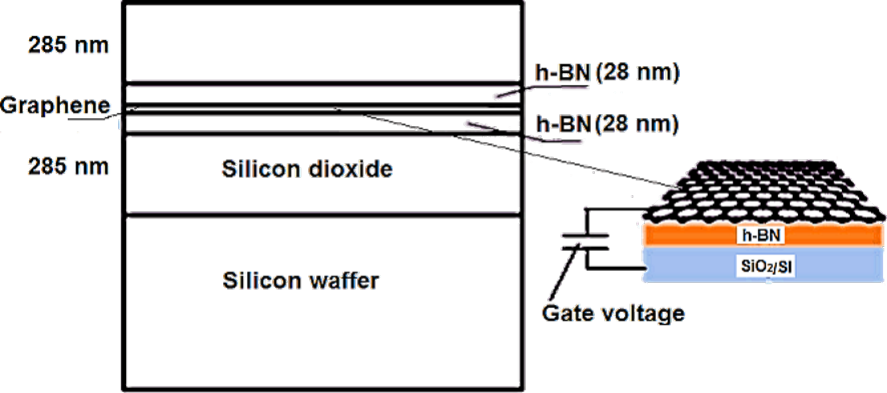
Graphene-based waveguide geometry.

The graphene/substrate geometry for a graphene-based waveguide in shown Figure 3. The use of a gate voltage provides the control of the graphene chemical potential. This value can operate in the conduction or valence band, depending on the applied voltage, due to the diffusion of negative particles (positive) in graphene (effect of the electric field) [21]. The increase of the gate voltage value causes the increase of the graphene conductivity value.

## Results and Discussion

Since the nanophotonic devices should operate in telecommunication networks, Gaussian pulses should be considered as the input: *A*(0,*t*) = *A ×* exp(−(*t*/τ_0_)^2^), where *A* is the pulse amplitude and τ_0_ is the temporal half width (i.e., where the amplitude is equal to *A* × 1/e). To obtain more reliable results, we also considered hyperbolic secant pulses (*A*(0,*t*) = *A* × sech(*t*/τ_0_)) in some cases.

The GSPP mode wavelengths and the propagation distance can be determined by its wave vectors as given by [22–25]:

[7]
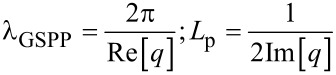


**Figure 4:**
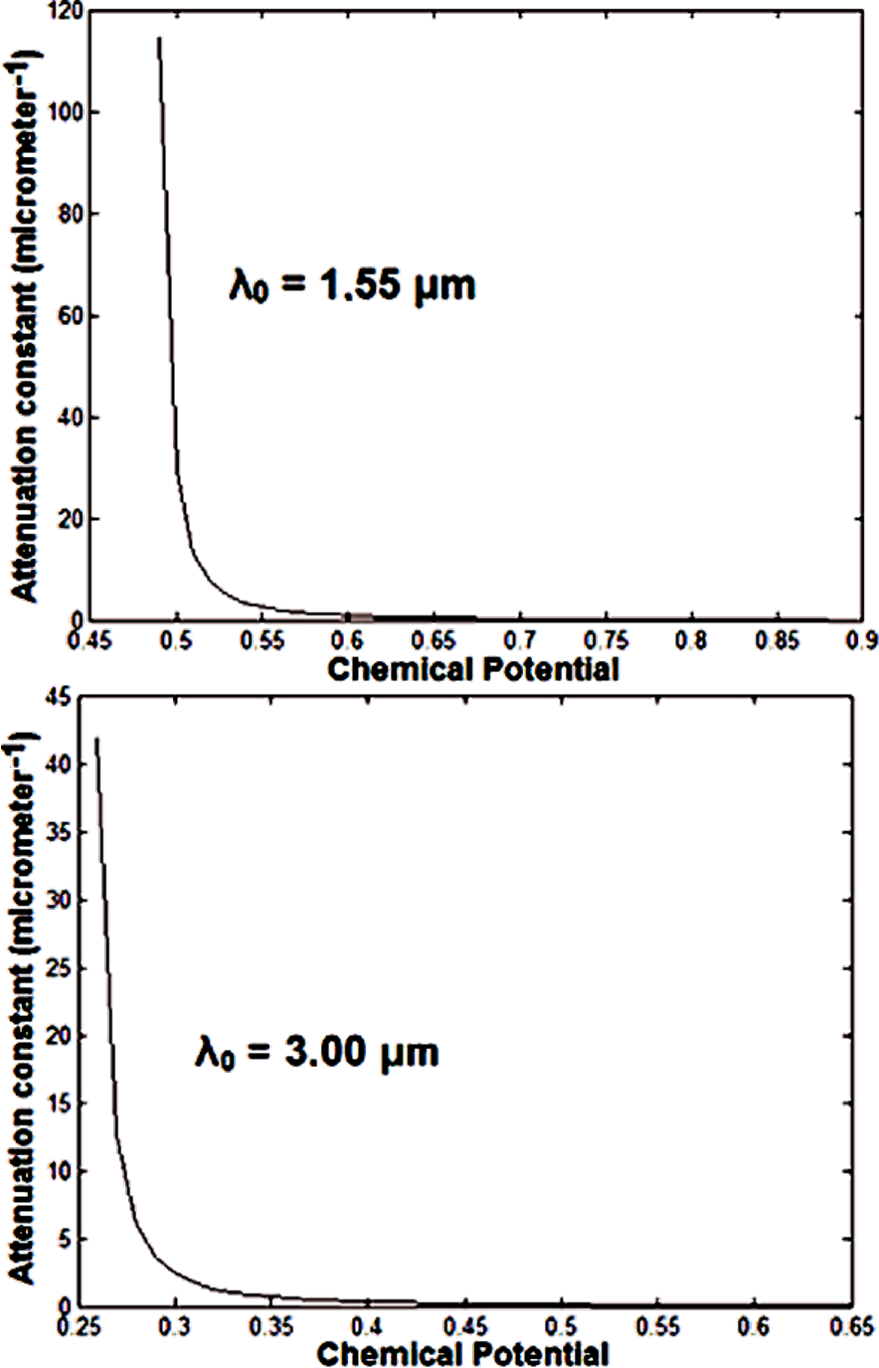
Attenuation constant (μm^−1^) versus chemical potential (for 1 *L_p_*).

Using Equation 7, we can find the propagation length corresponding to a GSPP mode propagating inside a graphene-based waveguide. The attenuation constant values (μm^−1^) referring to one propagation length, for λ_0_ = 1.55 μm and μ_g_ > 0.48 eV is illustrated in Figure 4 (top).

**Figure 5:**
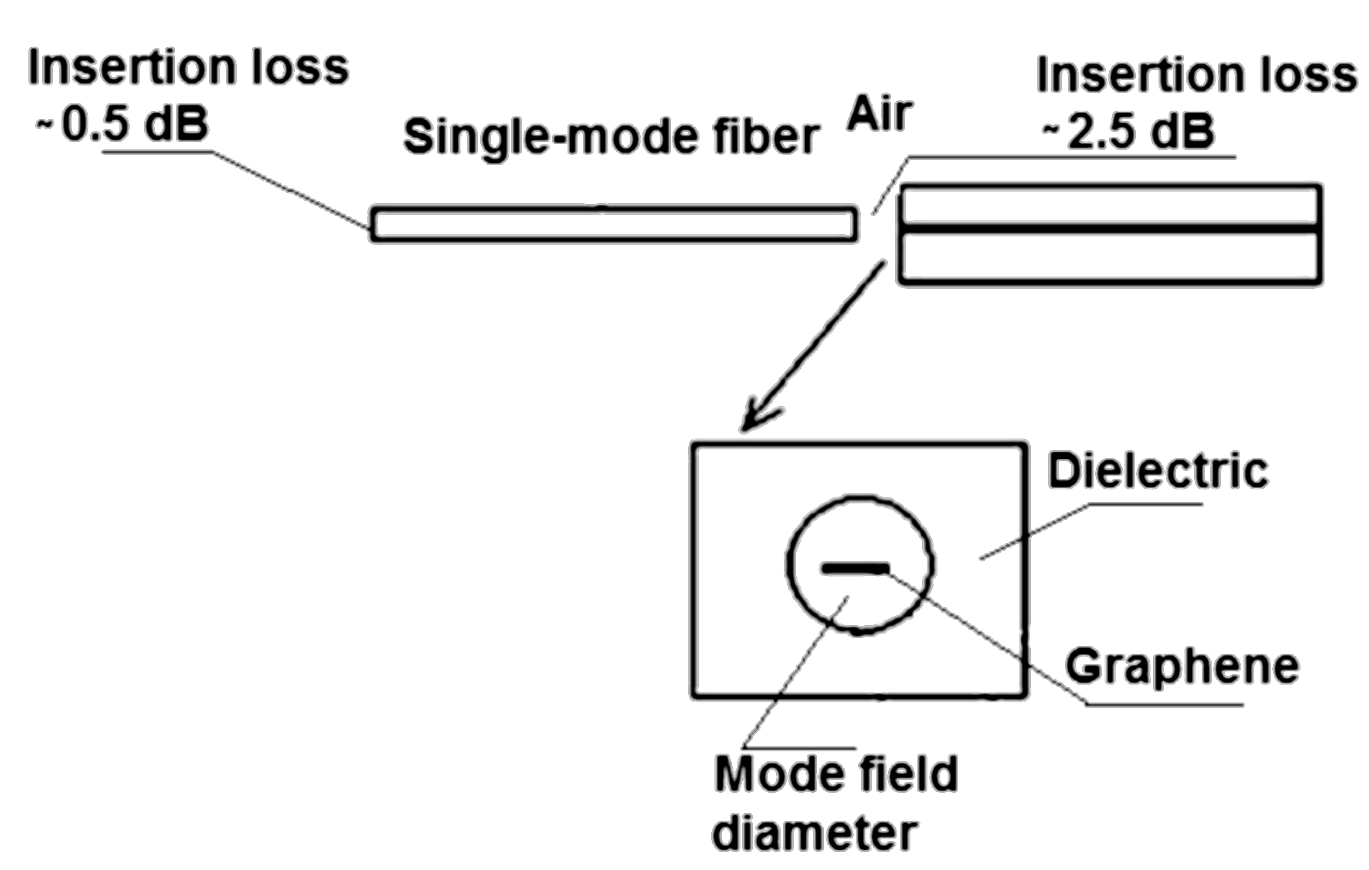
Schematic view of the insertion of an optical signal in a graphene-based waveguide.

Note that for μ_g_ ≈> 0.68 eV, the mode suffers virtually no attenuation. The attenuation constant values (μm^−1^) for one propagation length for λ_0_ = 3 μm and μ_g_ > 0.25 eV are given at the bottom of Figure 4. Note that in this case, for μ_g_ ≈> 0.43 eV, the mode suffers almost no attenuation. Therefore, since the graphene-based waveguide we are analyzing has a length smaller than 1 *L*_p_, we can neglect the attenuation inside the waveguide. However, we must consider the insertion loss. A schematic view for insertion of an optical signal in a nanophotonic waveguide is shown in Figure 5.

The optical signal from an optical source is inserted into a monomode optical fiber via a connector with an insertion loss of 0.5 dB, mainly due to misalignment between the two portions of the optical fibers. The insertion loss of the optical fiber can be neglected due to its short length. The signal output to be coupled to the graphene-based waveguide (Figure 5) suffers an estimated insertion loss of 2.5 dB (for safety) before entering the graphene nanoribbon due to misalignment and control of the proper distance between the optical fiber and the nanophotonic waveguide.

Considering that the modal area of the electromagnetic flux reaching the graphene nanoribbon center is approximately equal to the modal area of a single mode fiber, we can obtain the value of the intensity (*I*) at the input of the graphene-based waveguide. However, the electromagnetic flux coupled to the SPs (GSPPs) is subject to the electrical and optical graphene parameters. Therefore, the output pulse shape will suffer changes imposed by the graphene parameters.

**Figure 6:**
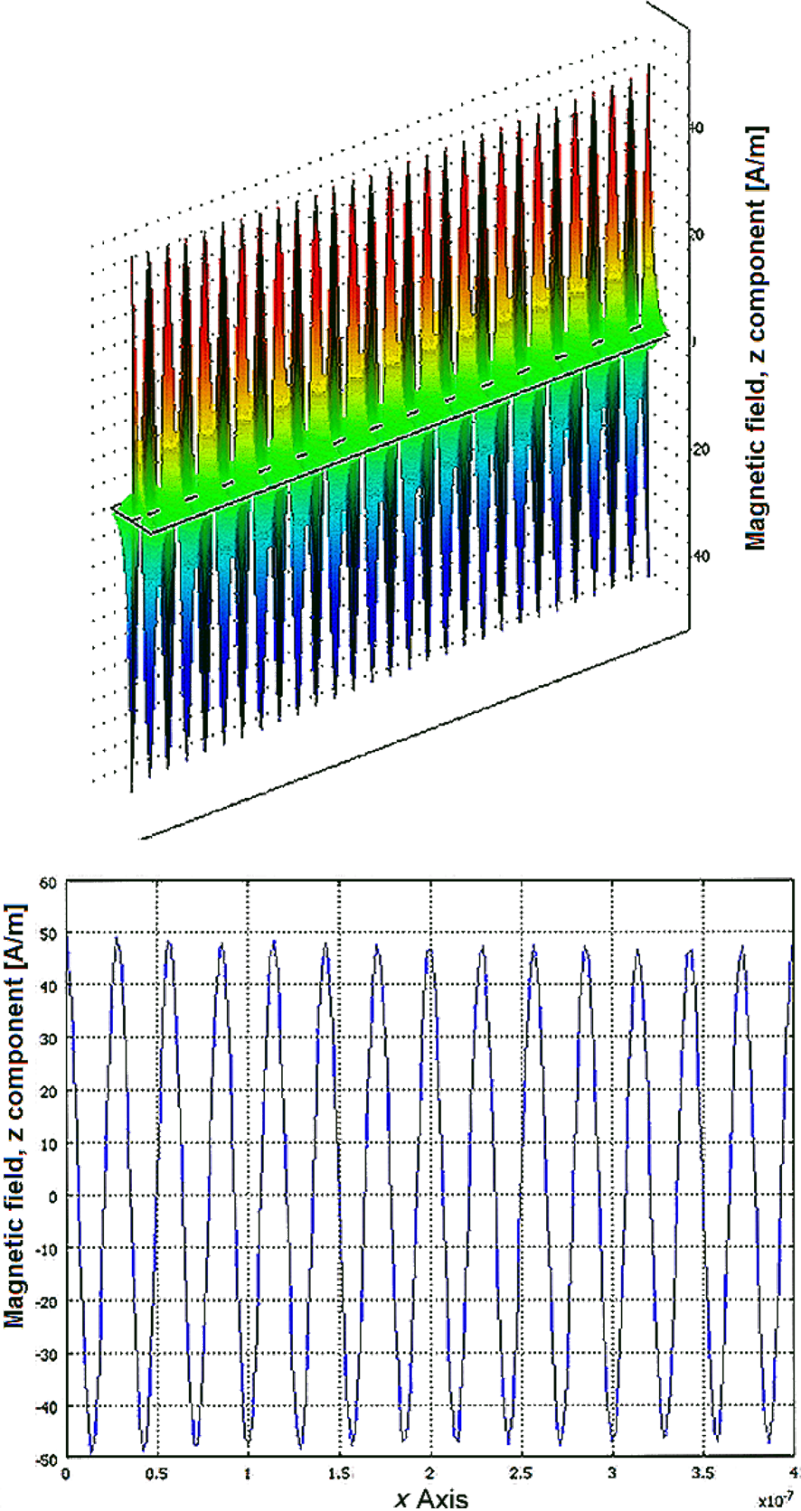
GSPP mode magnetic field pattern.

To prove that the attenuation can be neglected when the length of the graphene nanoribbon, *L*, is less than *L*_p_ we used COMSOL to determine the 3D magnetic field pattern of the GSPP propagating mode (*L* = 250 nm, λ_0_ = 3 μm, μ_g_ = 0.4 eV, σ = 6.786 × 10^−8^ + i5.282 × 10^−5^, ε_r_ = −26.945 + i0.036, *q* = 2.104 × 10^8^ + i2.702 × 10^5^), as shown in the top of Figure 6. A transverse cross section through the center of the naphotonic waveguide along its length is shown in the bottom of Figure 6, where we can see that the attenuation can be neglected for this waveguide length.

It is well known that for short pulses the dispersion and nonlinearity effect should not be considered individually, that is, it is necessary to consider the combined effects of dispersion and nonlinearity.

The behavior of the propagation of optical signals in a nonlinear medium can be determined by the following equation [26]:

[8]
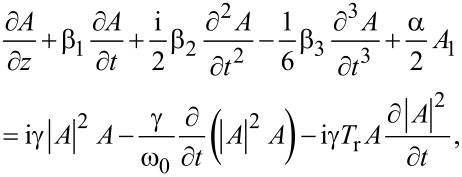


where the pulse amplitude (*A*) is normalized such that 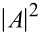
represents the intensity (*I*) of the propagating signal, β_1_ = 1/*V*_g_ is the parameter which determines the group velocity (*V*_g_), β_2_ is the second order dispersion parameter, β_3_ is the third order dispersion parameter, γ = *n*_2_ω_0_/*cA*_eff_ is the nonlinear coefficient (where *n*_2_ is the nonlinear refractive index, ω_0_ is the central angular frequency of the pulse, *c* is the speed of light, and *A*_eff_ is the effective area of the mode), α is the attenuation constant, and *T*_r_ ≈ 5 fs is a parameter related to the slope of the Raman gain. The second, third and fourth terms on the left side of Equation 8 determine the dispersion and attenuation effects. The three terms on the right side of Equation 8 are responsible for the spectral broadening of the pulse, self-steepening and shock formation and self-frequency shift, respectively.

In a previous work, the Fourier transform was applied to find the broadening of a pulse that propagates on a nanophotonic metal/dielectric waveguide. Here, a multiplication factor, *M*, for each component was inserted (in the frequency domain), which governs each frequency component with respect to the dispersive broadening, as the pulse propagates a certain distance [27]:

[9]
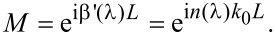


In Equation 9, β′ is the real part of the wave vector for each frequency component and *L* is the propagation distance (*L < L*_p_). After applying Equation 9, the inverse Fourier transform was performed to recover the time profile of the output signal. It is worth noting that during this procedure, the normalized intensity *I* is equal to 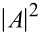.

We applied Equation 9 to find the temporal pulse broadening and included these values in Equation 8, substituting the second and the third term on the left side of that equation.

The advantage of using graphene as a nonlinear medium is the high value of its nonlinear refractive index *n*_2_ ≈ 10^−7^ cm^2^/W, which is much higher than the nonlinear refractive index of dielectrics typically used in optical communications (approximately 10^9^ higher) [28]. Moreover, the effective mode area was calculated as 2.2 × 10^−7^ × λ_0_^2^ [7], which is much smaller than the values obtained to date. In our calculations we used the effective graphene cross-section modal area.

The edge effects in a graphene nanoribbon appearing in the vicinity of the Fermi energy decrease as a function of the length of the nanoribbon and they are expected to become negligible at a nanoribbon length of a few micrometers [29]. Hence, taking into consideration that *L*_p_ = 4.146 μm (Equation 7), we can neglect the effects of the nanoribbon edges.

We considered the width, *W*, of the graphene nanoribbon to be 35 nm, which allows for only the fundamental mode to propagate through the nanophotonic waveguide. To simplify our calculations, we adopted a length of *L* = 500 nm < *L*_p_. Due to the large values of the nonlinear refractive index for graphene, as well as the modal intensity, nonlinear effects can appear, even for very small values of optical signal power.

**Figure 7:**
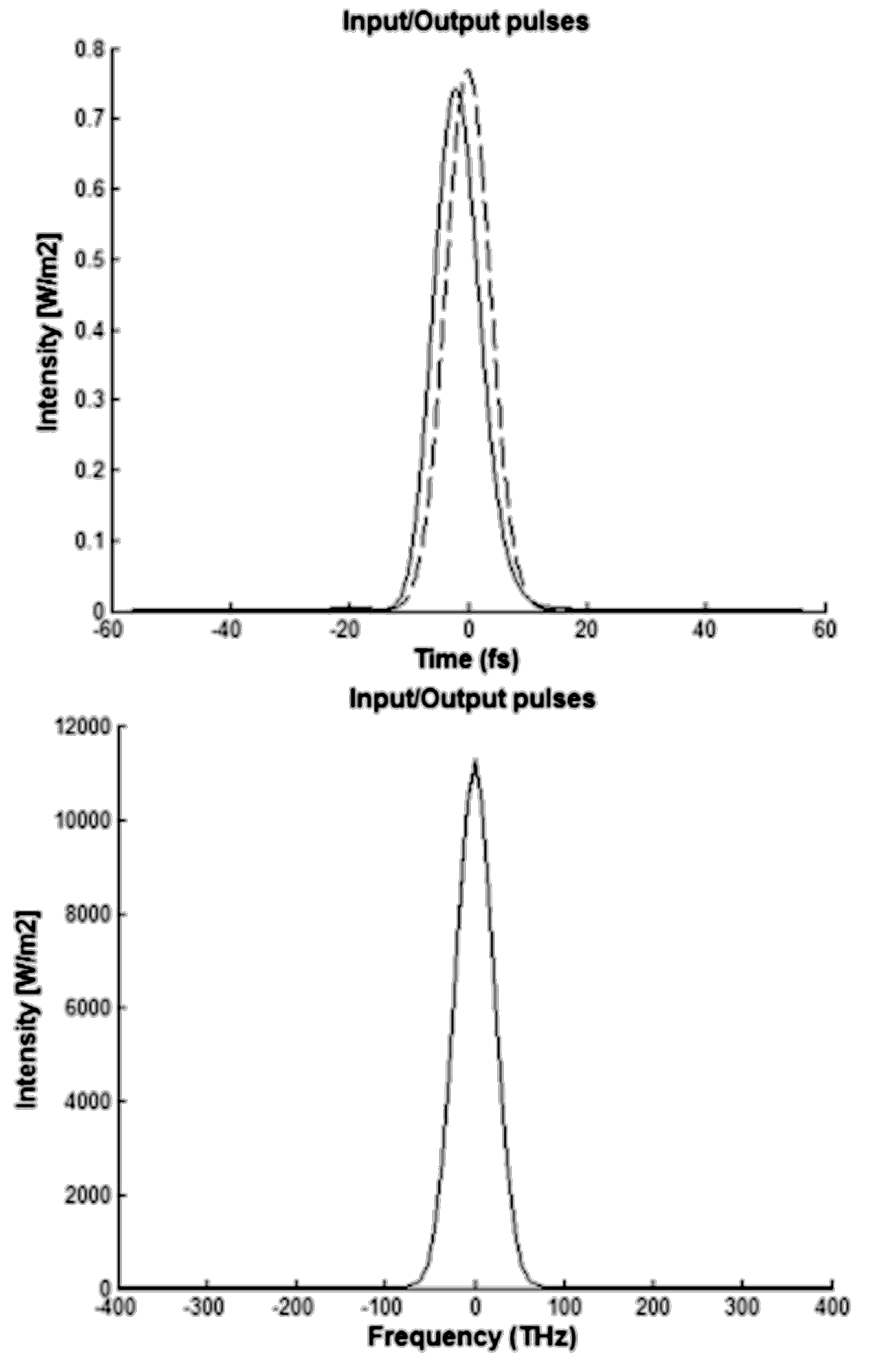
Gaussian pulse shape in the time domain (top) and frequency domain (bottom) for the nanophotonic waveguide (*P*_0_ = 10^−10^ W).

We show the intensity, which was calculated in accordance with Equation 8, relative to a fs Gaussian pulse (τ_0_ = 15 fs), as a function of time in the top part of Figure 7. The input pulse shape in the temporal domain is represented by a dashed line, and the output pulse shape in the temporal domain (after propagating *L* = 500 nm with an input signal power of *P*_0_ = 10^−10^ W, λ_0_ = 1.55 mm and μ_g_ = 0.80 eV) is represented by the solid line.

The intensity as a function of the frequency spectral component is shown at the bottom of Figure 7 where the input pulse shape in the frequency domain (dashed line) and the output pulse shape in the frequency domain (solid line) are shown after propagating *L* = 500 nm, with an input signal power *P*_0_ = 10^−10^ W, λ_0_ = 1.55 mm and μ_g_ = 0.80 eV. Notice that the shape of the Gaussian pulse in the time domain undergoes a small change, but the output Gaussian pulse in the frequency domain remains unchanged.

**Figure 8:**
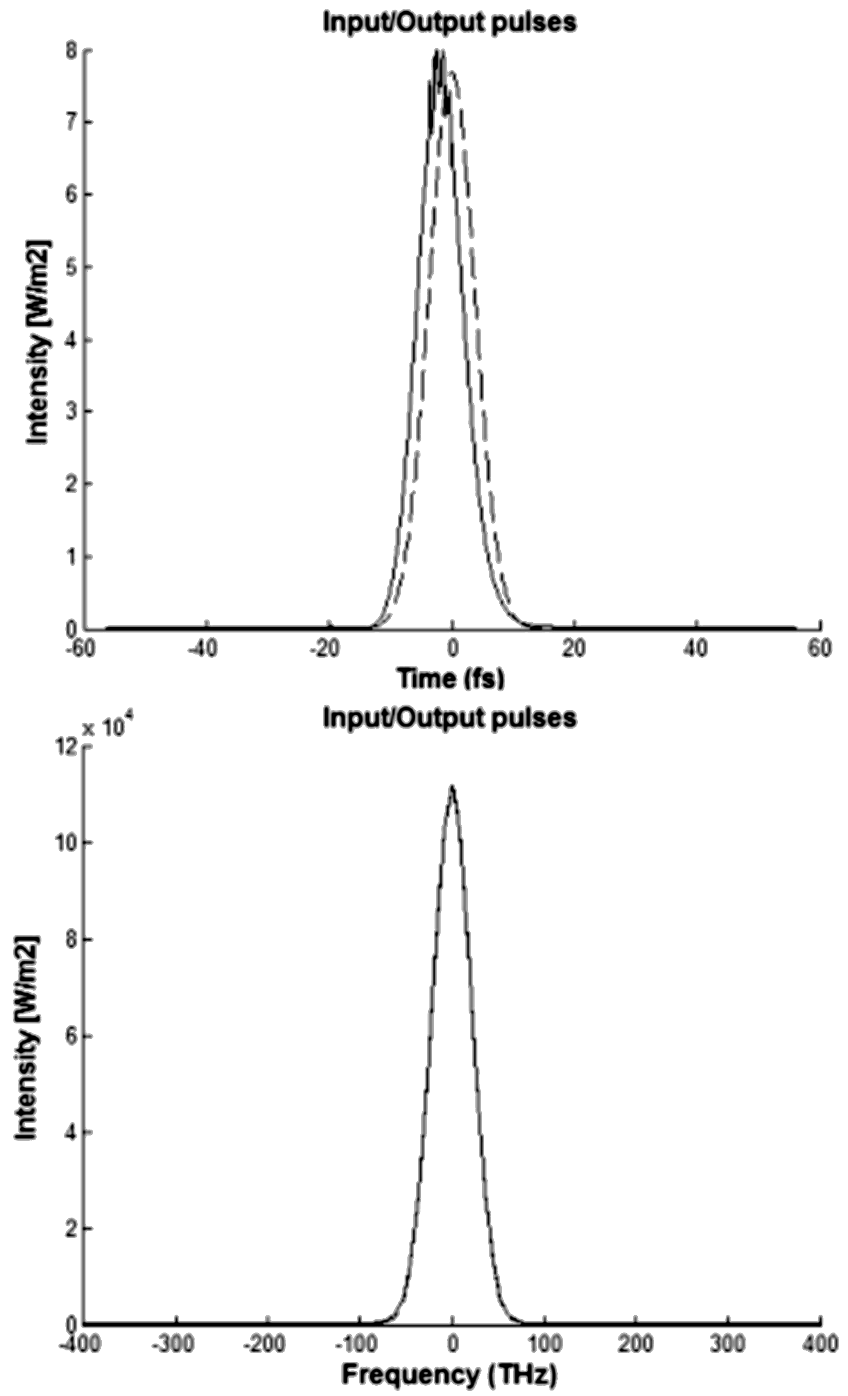
Intensity as a function of the time (top) and as a function of the frequency component (bottom) for the input and output pulses (*P*_0_ = 10^−9^ W).

In the upper part of Figure 8, the intensity, also calculated in accordance with Equation 8, is given as a function of time. The lower part of Figure 8 shows the intensity versus frequency spectral components related to Gaussian pulses with τ_0_ = 15 fs at the input (dotted line) and output (solid line) of the nanophotonic waveguide after propagating *L* = 500 nm, given an input signal power of *P*_0_ = 10^−9^ W (λ_0_ = 1.55 μm, μ_g_ = 0.80 eV). From Figure 8, one can see that these pulses with *P*_0_ = 10^−9^ W suffer a moderate change in the time domain, but they are not changed in the frequency domain.

After several simulations, we concluded that given the same propagation length, an increase in the signal power at the input results in strong nonlinear and dispersive effects, which distort the shape of the Gaussian pulses in the time and frequency domain at the output. Additionally, it was found that increasing the value of the chemical potential of the graphene resulted in dispersive effects that distort the shape of the Gaussian pulse in the time domain at the nanophotonic waveguide output.

**Figure 9:**
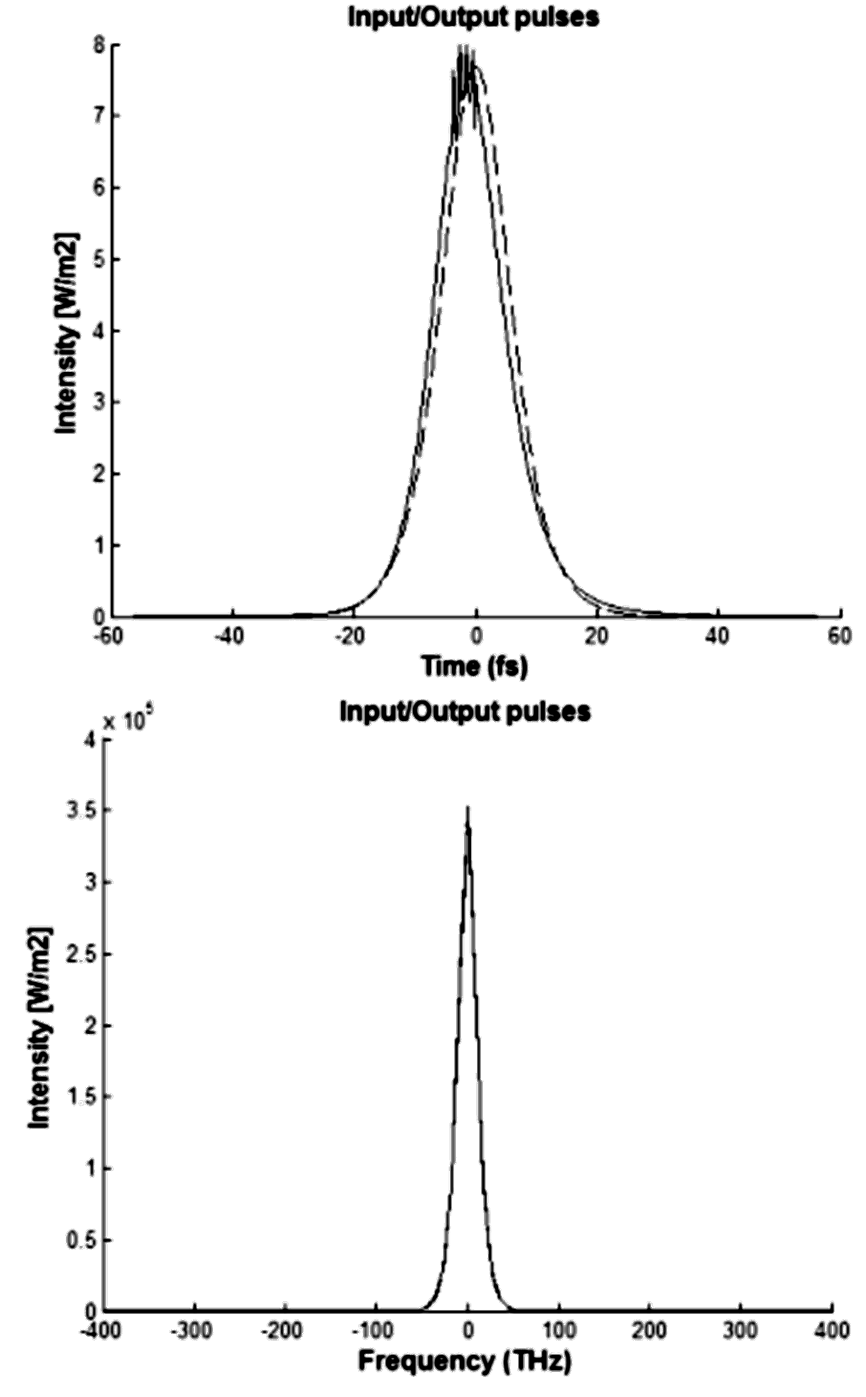
Hyperbolic secant pulse shape in the time domain (top) and frequency domain (bottom) for the input and output pulses (*P*_0_ = 10^−9^ W).

To conclude our simulations regarding graphene-based nanophotonic waveguides, we used a hyperbolic-secant-shaped pulse in the time domain. The results of the intensity (calculated according to Equation 8) as a function of time at the input (dotted line) and at the output (solid line) of the nanophotonic waveguide are shown at the top of Figure 9, after the pulse propagates over a length *L* = 500 nm for the input signal power *P*_0_ = 10^−9^ W (λ_0_ = 1.55 μm, μ_g_ = 0.80 eV). Shown at the bottom of Figure 9 are the results of the intensity calculated from Equation 8 as a function of frequency at the input (dotted line) and the output (solid line) of the nanophotonic waveguide. Similar to the results using a Gaussian pulse, the hyperbolic-secant-shaped pulse is also moderately changed in the time domain but not in the frequency domain.

## Conclusion

In this work we have shown the effects related to the attenuation, high-order dispersion and high-order nonlinearity in graphene-based nanophotonic waveguides. In particular, the changes in the shape of ultrashort Gaussian and hyperbolic secant input pulses due to the attenuation, dispersion and nonlinearities were analyzed. We concluded that it is possible to control the shape of the output pulse as a function of the input signal power and chemical potential of graphene. We believe that the obtained results will be very relevant to the development of graphene-based nanophotonic devices, such as filters, modulators, antennas, switches, and other devices.
